# Natural history of SPP1 signaling in NF1 tumors

**DOI:** 10.1038/s41698-025-01078-2

**Published:** 2025-10-06

**Authors:** Kimani Njoya, Huda Zayed, Li Sun, Donia Alson, Oluwatosin Aina, Sajjad Khan, Ximei Veneklasen, Nikki Lytle, Pradeep Chaluvally-Raghavan, Daochun Sun

**Affiliations:** 1 Department of Cell Biology, Neurobiology, and Anatomy, Medical College of Wisconsin, Milwaukee, WI USA; 2 Department of Surgery, Medical College of Wisconsin, Milwaukee, WI USA; 3 Department of Obstetrics and Gynecology, Medical College of Wisconsin, Milwaukee, WI USA; 4 Department of Pediatrics, Medical College of Wisconsin, Milwaukee, WI USA; 5 Children’s Research Institute, Children’s Wisconsin, Milwaukee, WI USA

**Keywords:** Cancer, Cancer microenvironment, Cancer stem cells, Tumour heterogeneity

## Abstract

Understanding the heterogeneity of Neurofibromatosis type 1 (NF1)-associated tumors and delineating the natural historical evolution of cell signaling are essential for interpreting tumor initiation, preventing tumor progression from benign plexiform neurofibromas (pNFs) to malignant peripheral nerve sheath tumors (MPNSTs), and engineering effective treatments. The neural crest-derived Schwann cell precursor (SCP)-like tumor population interacts with different cells in the tumor microenvironment (TME), particularly macrophages, continually shaping the intrinsic and extrinsic NF1 tumor heterogeneity. Through integrated analyses of single-cell RNA-seq (scRNA-seq) and spatial transcriptomics, we reveal that SPP1-CD44 signaling is initiated by SCP-like tumor cells in pNF, operating through autocrine mechanisms. However, in MPNST, a distinct subset of macrophages becomes the dominant SPP1 signaling source while the SCP-like cells maintain autocrine signaling. The role of SPP1 in tumorigenesis is validated by the significantly extended survival in the MPNST mouse model with *cisNf1*^*+/-*^*;Trp53*^*+/-*^*;Spp1*^*-/-*^ configuration. Notably, our analysis of the pre-tumor stage in the *DhhCre;Nf1*^*-/-*^ pNF mouse model demonstrates upregulated *Spp1* expression compared to control tissue in *Nes*^*+*^ Schwann lineage cells. Together, these findings elucidate the natural historical dynamics of SPP1-CD44 signaling during tumor initiation and progression from pNF to MPNST, and highlight the SPP1-CD44 signaling axis as a potential therapeutic target to disrupt tumor stemness properties and reprogram the immune TME in malignancies.

## Introduction

Neurofibromatosis type 1 (NF1) is a monogenic disorder affecting 1:2500 newborns. NF1 patients have high vulnerability to peripheral nervous system (PNS) and central nervous system (CNS) tumors, such as plexiform neurofibroma (pNF) and low-grade glioma (LLG). pNFs are benign PNS tumors frequently observed in pediatric populations and have a high chance of progressing to malignant peripheral nerve sheath tumors (MPNSTs)^1^. pNF and MPNST are heterogeneous, with complex and dynamic intercellular crosstalk within the tumor microenvironment (TME). The extrinsic tumor heterogeneity was described using immunohistochemistry staining^2^ and further delineated with single-cell-based technologies^3–6^. Myeloid lineage infiltration, especially an abnormally high number of macrophages, in the TME is intriguing, and the evolving signal crosstalk concurrent with tumor-initiating, immune cell infiltration, and tumor progression requires further investigation^7^. NF1-associated pNFs and MPNSTs originate from Schwann cell lineage (SCL) cells that arise from neural crest (NC) cells^8,9^. NC cells differentiate into Schwann cell precursors (SCP), glial progenitors that express key markers, such as *NES* and *ERBB3*^10,11^. SCP further differentiates into immature Schwann cells, non-myelinating Schwann cells, and myelinating Schwann cells, which are mediated by NRG1-ERBB3 signaling and other factors^12,13^. In the absence of proper NF1 function in the early SCL, the developmental force and sustained inflammation, compounded by the stem cell properties, promote pNF formation. The SCP-like tumor cells have been identified and characterized in human and mouse tumor models; however, the mechanism for sustaining the stem cell properties is still unclear^4,5,9^.

The secreted phosphoprotein 1 (SPP1), also known as Osteopontin, has garnered considerable interest due to its multifaceted role in various immune and stromal cell interactions within TME^14^. SPP1 is expressed by various cell types, including osteoblasts, fibroblasts, macrophages, and dendritic cells^15–19^. In physiological contexts, SPP1 is involved in bone development, tissue repair, and extracellular matrix formation^20,21^. Aberrant expression of SPP1 is associated with pathological fibrotic processes, chronic inflammation, vascular calcification, tumor formation, and metastasis^22–25^. SPP1 function is mediated by binding to CD44 and several integrins. CD44 is particularly significant as it has been reported as a marker of cancer stem cells in breast, gastric, and pancreatic cancers, with roles in tumor initiation and maintenance of stem-like properties^26^. CD44 expression has also been demonstrated as a signature of NC stem cell-derived tumors, including melanoma^27,28^, Schwannoma, pNF and MPNST, and contributes to aggressive phenotypes^29,30^. SPP1-CD44 axis has been shown to enhance the self-renewal and maintenance of cancer stem cells and sustain the malignant phenotype in solid tumors, properties that are pivotal for tumor progression^31–33^. However, the dynamics of SPP1 signaling in NF1 tumorigenesis and progression remain unclear. Characterizing the cell types involved in SPP1 signaling during NF1 tumorigenesis is essential for comprehensively understanding the role of SPP1 and engineering a timely treatment.

## Results

### SPP1 promotes nervous system tumors

**Fig. 1:**
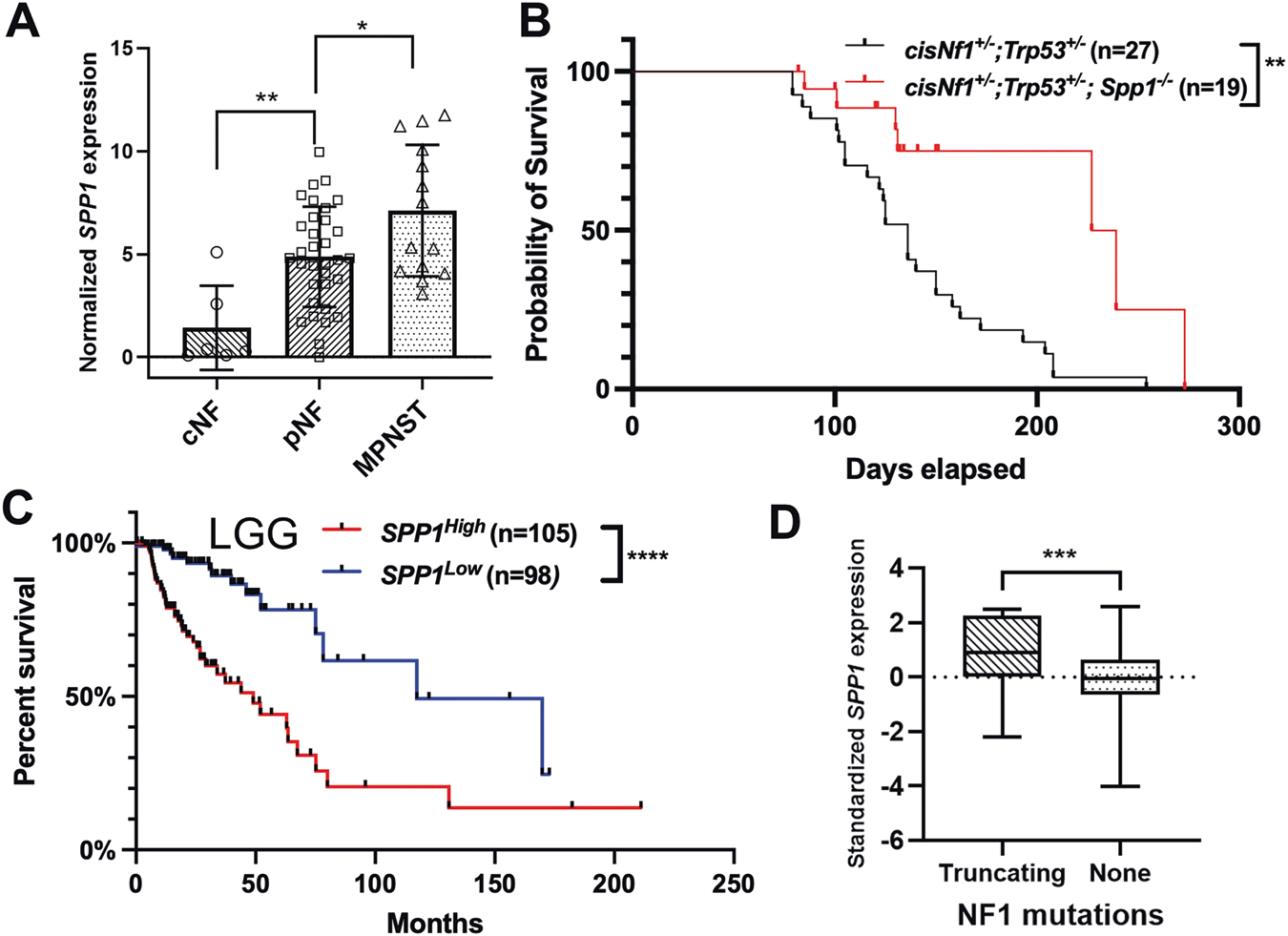
SPP1 promotes the progression of NF1-associated tumors in the peripheral and central nervous systems. **A** Normalized *SPP1* expression from bulk RNA-seq of human cNFs, pNFs and MPNSTs. **B** *Spp1* knockout expands the survival of spontaneous MPNST mouse models. **C** Human LGG dataset shows survival stratification between *SPP1*^*high*^ and S*PP1*^*low*^ groups. **D** LGG patients with NF1 truncating mutation have significantly higher expression of *SPP1*. *p < 0.05, **p < 0.01, ***p < 0.001, ****p < 0.0001.

SPP1 expression is increased in human MPNSTs compared to pNF, and the knockdown of SPP1 in MPNST cell lines led to reduced spheroid formation and migration in vitro^34^. Wu et al. also indicated that SPP1 signaling is activated in both pNF and MPNST based on single-cell RNA sequencing (scRNAseq) analysis^4^. To systematically assess the relationship between SPP1 expression and NF1 tumor progression, we examined a bulk RNA sequencing (RNA-seq) dataset containing 54 NF1-associated tumors, including 6 cutaneous neurofibromas (cNFs), 32 pNFs, and 13 MPNSTs curated in Cbioportal^35,36^. SPP1 expression correlates with tumor severity, from cNF to MPNST (Fig. 1A). To assess the functional impact of SPP1 on survival outcome, we crossed *Spp1*^*-/-*^ mice with the spontaneous MPNST mouse model, *cisNf1*^*+/-*^*;Trp53*^*+/-*^^37^. A comparison of survival rates showed a significantly extended survival of the *cisNf1*^*+/-*^*;Trp53*^*+/-*^*; Spp1*^*-/-*^ than *cisNf1*^*+/-*^*;Trp53*^*+/-*^ mice, indicating that SPP1 plays a crucial role in the progression of NF1-associated MPNST (Fig. 1B).

To evaluate the impact of *SPP1* expression on patient survival, we adapted a low grade glioma (LGG) study curated by Cbioportal, given the lack of pNF or MPNST datasets with transcriptomics and patient survival information. Loss-of-function mutation of *NF1* is reported in 15–20% of glioma patients^38^. Furthermore, pediatric LGGs arise from the glial lineage in the central nervous system and occur in up to 20% of children with NF1^39^. Bulk RNA-seq and clinical data from 632 LGG patients were downloaded and analyzed^40^. The LGG samples were stratified by standardized *SPP1* expression into *SPP1*^*high*^ (Expression > 0.8, ~top 20% patients) and *SPP1*^*low*^ (Expression < −0.8, ~bottom 20% patients). Log-rank-based survival analysis demonstrated significantly worse survival in the *SPP1*^*high*^ cohort than the *SPP1*^*low*^ cohort (Fig. 1C).

Interestingly, gene mutation analysis on 632 LGGs reveals that patients with *NF1* truncating mutations exhibit significantly higher *SPP1* expression than those without *NF1* mutations (Fig. 1D). This suggests that the loss of *NF1* is strongly concomitant with increased *SPP1* expression in LGG tumor tissue. Cbioportal oncoprint also showed that *NF1* mutations are enriched in the *SPP1*^*high*^ LGG samples and are almost excluded from the *SPP1*^*low*^ patients (Supplementary Fig. 1). These findings prompted us to characterize the complex TME in NF1-associated tumors to track the temporal and spatial evolution of SPP1 signaling.

### SPP1 secreted from SCP-like tumor cells

**Fig. 2:**
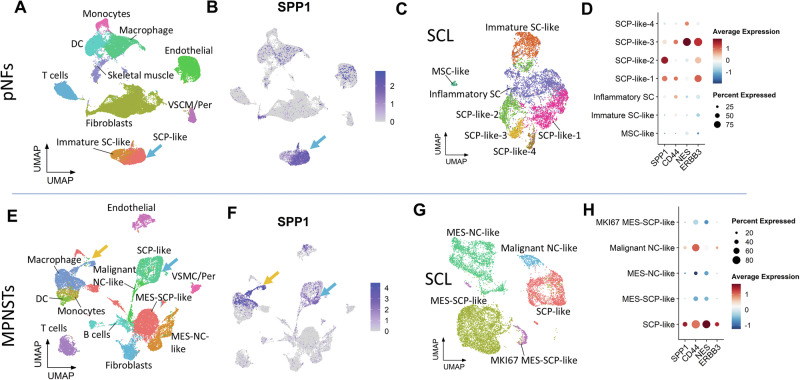
SPP1-CD44 signaling is enriched in the stem-like SCL populations. **A** scRNAseq clusters of integrated human pNFs in UMAP. **B** UMAP plot of SPP1 expression in pNFs. The blue arrow indicates the SCP-like cluster. **C** Subclustering shows different cell clusters from the SCL in pNFs. **D** Dot plots of SPP1, CD44, NES and ERBB3 expression in pNF SCL clusters. **E** scRNAseq clusters of integrated human MPNSTs in UMAP. **F** UMAP plot of SPP1 expression in MPNSTs. The blue arrow indicates the SCP-like cluster, and the yellow arrow indicates the macrophage cluster. **G** Subclustering shows different cell clusters from the SCL in MPNSTs. **H** Dot plots of SPP1, CD44, NES and ERBB3 expression in MPNST SCL clusters. MES Mesenchymal; NC neural crest.

To investigate the dynamics of SPP1 signaling in human pNFs and MPNSTs, we analyzed scRNAseq data from the Wu et al. study^4^ and adopted cell identities from their study. A total of 50,983 cells were integrated from 10 pNF samples and visualized using UMAP (Fig. 2A). Notably, *SPP1* expression is enriched in SCP-like cells, with lower expression observed in macrophages, endothelial cells, and a subset of fibroblasts (Fig. 2B and Supplementary Fig. 2A). Further subclustering of the SCL cells revealed that SCP-like-1 co-expresses *SPP1*, *NES*, and *ERBB3* (Fig. 2C, D and Supplementary Fig. 2B). A total of 20,301 cells from four human MPNSTs were integrated as shown in Fig. 2E. The SCL populations in MPNSTs showed greater heterogeneity compared to pNF, with the emergence of MES-SCP-like, MES-NC-like and Malignant NC-like clusters (Fig. 2E and Supplementary Fig. 2C). In contrast to pNFs, *SPP1* is highly expressed in macrophages and modestly in the SCP-like cluster in MPNSTs (Fig. 2F). Clustering of SCL cells further delineated the subgroups (Fig. 2G and Supplementary Fig. 2D). *SPP1* expression is conserved in SCP-like tumor cells that maintain the expression of *NES, ERBB3*, and, notably, *CD44* (Fig. 2H). However, tumor clusters with mesenchymal features have minimal *SPP1* and *CD44* expression. These observations indicate that SPP1 signaling is involved in the early tumorigenic process starting from the benign tumor stage and may play roles in maintaining the stemness of tumor cells, programming macrophages and promoting tumor transformation via the SPP1-CD44 axis^31,32,41^.

### SPP1 secreted from macrophages in MPNST TME

**Fig. 3:**
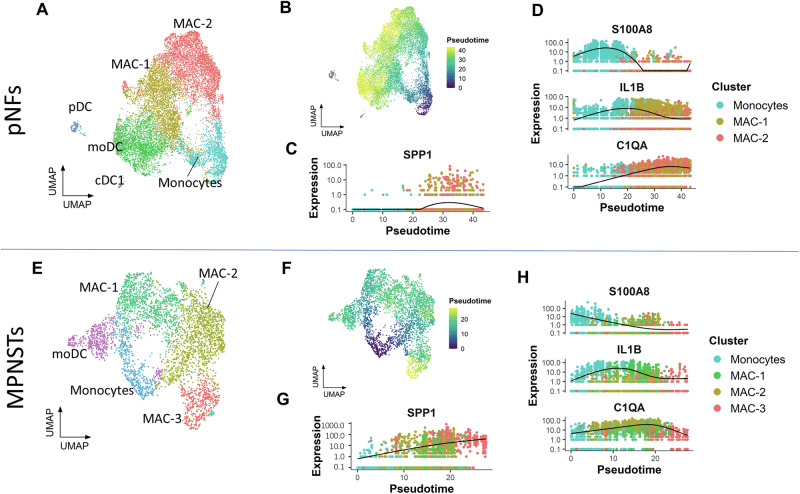
Myeloid lineage differentiation in the TME of NF1 tumors. **A** Subclustering of myeloid lineage in pNFs. **B** Pseudotime analysis of myeloid lineage differentiation in pNFs. **C** *SPP1* expression in pNF myeloid cells along the pseudotime. **D** *S100A8*, *IL1B*, and *C1QC* expression along the pseudotime. **E** Subclustering of myeloid lineage in MPNSTs. **F** Pseudotime analysis of myeloid lineage differentiation in MPNSTs. **G** SPP1 expression in MPNST myeloid cells along the pseudotime. **H** *S100A8*, *IL1B*, and *C1QC* expression along the pseudotime in MPNSTs. pDC plasmacytoid dendritic cells; moDC monocyte-derived dendritic cells; cDC1 conventional dendritic cells type 1.

The myeloid lineage, including monocytes and macrophages, is among the most abundant immune cell populations in NF1-associated pNFs and MPNSTs (Fig. 3A, E and Supplementary Fig. 3). Macrophages are a highly plastic cell type derived from monocytes and phenotypically classified into M1 and M2 phenotypes to emphasize their classically activated and alternatively activated phenotypes^42^. However, their roles in tumorigenesis are highly customized according to the tumor type and scenario^43^. In the TME of pNF and MPNST, infiltrated monocytes and tumor-associated macrophages (TAMs) can be programmed into different phenotypes (Fig. 3A, B). The SPP1-expressing macrophages have been reported in TME of other malignant tumors, such as glioblastoma (GBM,) lung cancer, and breast cancer^44,45^. To understand the temporal dynamics of SPP1 expression in the monocyte to macrophage differentiation trajectory, pseudotime analysis was performed using Monocle3 (Fig. 3B). *SPP1* expression was gradually elevated during the differentiation of monocytes in pNF. *SPP1* is sparsely expressed in macrophage (MAC)-1 but slightly increased in MAC-2 (Fig. 3C). While the monocyte marker *S100A8* decreases, the M1 marker *IL1B* increases, and then the M2 marker *C1QC* increases (Fig. 3D). Interestingly, the clustering of MPNST myeloid cells reveals an additional macrophage cluster, MAC-3 (Fig. 3E). The pseudotime analysis demonstrated a trajectory from monocyte to MAC-1, MAC-2 and MAC-3. Notably, *SPP1* is consistently elevated and eventually enriched in MAC-3 (Fig. 3F, G). Meanwhile, the trend in expression of *S100A8*, *IL1B*, and C1QC along pseudotime is similar to pNFs (Fig. 3D, H). The high macrophage *SPP1* expression in MPNSTs is consistent with the reports of other malignancies, and the characterization of MAC-3 highlights the pro-tumor function of SPP1 signaling for its essential role during tumor progression.

### SPP1-CD44 dynamics in NF1 tumor progression

**Fig. 4:**
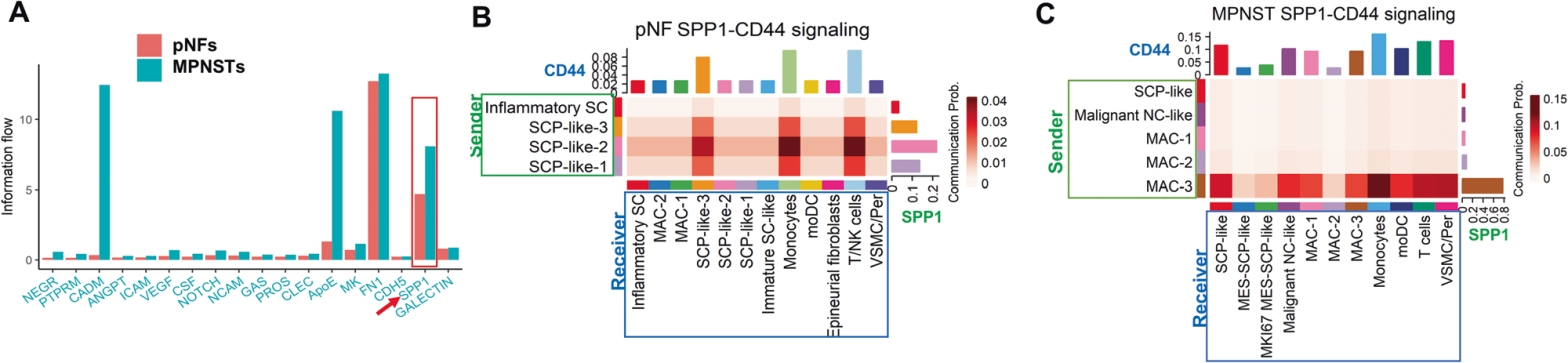
SPP1 signaling dynamics in NF1 tumor progression. **A** CellChat-based signaling comparison between pNFs and MPNSTs. Information flow represents the sum of communication probabilities between all pairs of cell clusters in each signaling pathway. **B** SPP1-CD44 signaling in pNFs (only cell types with significant signaling interactions are shown in the heatmap). Clusters with significant SPP1 expression were labeled as Sender (green framed), and clusters with significant CD44 expression were labeled as receiver (blue framed). **C** SPP1-CD44 communications in MPNSTs. The top bar graphs in each heatmap show the total incoming signaling strength for each cluster while the bar graphs on the right show total outgoing signaling strength for each cluster.

In pNFs tumor cells, both *SPP1* and *CD44* are expressed in the SCP-like cluster (Fig. 2D), suggesting SPP1 may function in an autocrine manner to sustain the stemness of the tumor-initiating cells. In MPNSTs, with increased expression of *SPP1* in the myeloid lineage, especially MAC-3, *CD44* expression was also elevated in the SCP-like cluster (Fig. 2H). This reinforced pattern is supported by the increased total SPP1 signaling intensity from pNFs to MPNSTs in an integrated comparison (Fig. 4A). SPP1-CD44 signaling influences the phenotypes of immune cells, including monocytes, macrophages and NK/T cells, which can lead to a pro-tumorigenic microenvironment^31,46^.

CellChat was employed to assess the SPP1-CD44 intercellular signaling among different cell types using the same dataset in Fig. 3^47^. In pNFs, the significant signal senders of SPP1 are SCP-like clusters, and the top significant receivers are SCP-like-3, monocytes, and T/NK clusters (Fig. 4B). The senders and receivers were determined by the CellChat algorithm as the significant contributors of SPP1-CD44 signaling. While SCP-like are the top senders in pNF, MAC-3 becomes the dominant sender in MPNST (Fig. 4C). SCP-like clusters remain the dominant receivers within the SCL population, with other top receivers including monocytes, macrophages, T cells, and vascular smooth muscle cells/pericytes (VSMC/Per). Interestingly, pseudotime analysis showed that MAC-3 is the latest developed macrophage subtype, indicating that MAC-3 may be programmed in the malignant TME and adapt to the high SPP1 expression (Fig. 3F, G). SPP1 has been shown to promote the differentiation of monocytes into alternatively activated macrophages. During progression, SPP1 may promote a self-reinforcing mechanism in monocyte differentiation and macrophage programming. The key question is where the original aberrant SPP1 comes from.

### The *Nes* + SCL expresses SPP1 at the pre-tumor stage

**Fig. 5:**
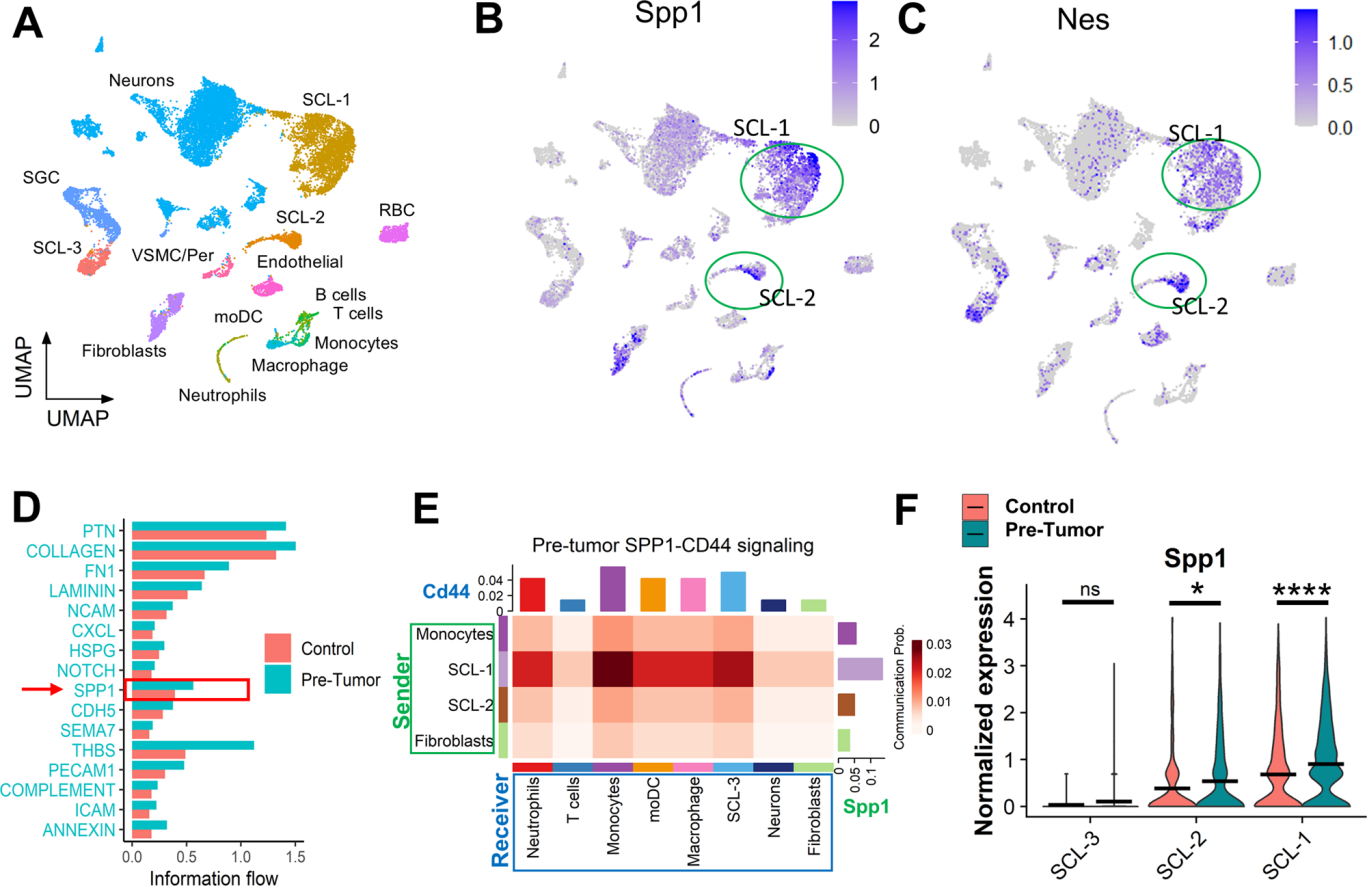
*Nes*-positive SCL clusters enrich *Spp1* expression in scRNAseq data of two-month-old dorsal root ganglia from *DhhCreNF1*^*f/f*^ mice and controls. **A** UMAP representation of the integrated two-month-old pre-tumor and age-matched control DRGs. **B** *Spp1* and **C** *Nes* expression is enriched in SCL-1 and SCL-2 (green circles) in pre-tumor DRGs. **D** CellChat-based signaling comparison between the pre-tumor and control. SPP1 signaling is indicated by the red arrow. **E** SPP1-CD44 communication in pre-tumor microenvironment. Clusters with significant *Spp1* expression were labeled as Sender (green framed), and clusters with significant *Cd44* expression were labeled as Receiver (blue framed). *Cd44* *Spp1* **F** Comparison of *Spp1* expression in the SCL clusters between pre-tumor and control DRGs. Wilcoxon Rank Sum Test (*p < 0.05, ****p < 0.0001).

To characterize SPP1 signaling during pNF tumorigenesis, we analyzed scRNA-seq data from 2-month-old *DhhCre;Nf1*^*f/f*^ mice^3^. This model develops pNFs at 8 months old, commonly located at the dorsal root ganglia (DRG) in the upper thoracic or cervical spinal regions. The 2-month-old pre-tumor tissue and the control tissue were harvested from the frequently tumor-forming DRGs, and then subjected to scRNAseq analysis^3^. Three pre-tumor tissue samples were integrated, and the UMAP revealed three SCL clusters (SCL-1, SCL-2, and SCL-3), fibroblasts, neurons, satellite glial cells (SGCs), and much less infiltrated immune cells (Fig. 5A). While the pre-tumor and control DRG share most cell types, the cell numbers of SCL-1, macrophages, and fibroblasts are much higher in the pre-tumor. Interestingly, *Spp1* is dominantly expressed by SCL-1 and SCL-2 (Fig. 5B), which co-express *Nes* (Fig. 5C). *Nes* has been indicated as a marker for stem-like tumor populations in studies of CNS and PNS tumors, such as glioblastoma and NF1-associated MPNST. The consistent expression of *Nes* from E13.5 neural crest stem cells to the SCP-like tumor cells delineate a natural historical progression path for MPNST tumorigenesis in the mouse model^5,48–50^. CellChat analysis demonstrated increased SPP1 signaling in pre-tumor compared to the control (Fig. 5D). In the SPP1-CD44 signaling axis, the high *Spp1-expressing* SCL-1, SCL-2, monocytes, and fibroblasts are among the senders (Fig. 5E) and the *Cd44*-expressing cell types within the pre-tumor microenvironment, especially the monocytes and SCL-3 are the dominant receivers (Fig. 5E). These interactions may prepare the microenvironment for pNF formation. Notably, SCL-1 in the pre-tumor has significantly higher expression of *Spp1* compared to the control (Fig. 5F). The aberrant increase of *Spp1* from *Nes* + SCL cells at the pre-tumor stage is likely an initiating event of the self-reinforced *Spp1* expression pattern in the microenvironment.

### SPP1-CD44 crosstalk in spatial proximity in human pNFs

TME intercellular crosstalk can be predicted by CellChat analysis; however, the signaling can be limited by physiological conditions and the proximity of the different cell types. To evaluate the feasibility of SPP1-CD44 interaction in pNF, we used the spatial RNA sequencing dataset of a human pNF^6^. The data were generated on the 10X Visium platform, with each capture spot representing transcripts from approximately 5–10 cells on the tissue section. While resolution is not at the single-cell level, the spatial transcriptome reflects the average gene expression within each defined tissue area. We hypothesized that spots enriched for SCP-like and MAC-2 signatures would be the prominent sources of SPP1, and that the SCP-like population, co-expressing *NES*, *ERBB3*, and *CD44*, should be spatially co-localized with *SPP1*-expressing cells, enabling the SPP1-CD44 autocrine or paracrine signaling.

**Fig. 6:**
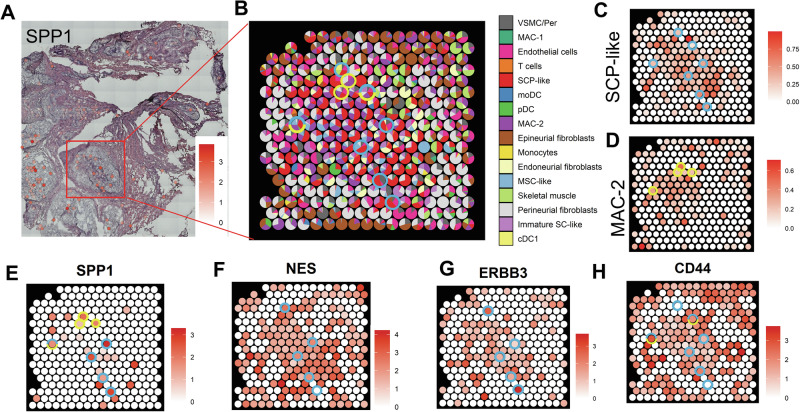
pNF spatial transcriptome reveals the autocrine and paracrine of SPP1-CD44 signaling in TME. **A** *SPP1* expression in pNF tissue. **B** Deconvolution of the spatial transcriptome using scRNAseq signatures in the selected tissue area. Turquoise and yellow circles indicate selected spots with dominant SCP-like and MAC-2 expressions, respectively. **C** SCP-like enriched spatial spots. **D** MAC-2 enriched spatial spots. **E** *SPP1*, **F** *NES*, **G** *ERBB3*, and **H** *CD44* expression in the spatial analysis.

We characterized *SPP1* expression in a human pNF dataset (Fig. 6A). Cell type deconvolution was then performed using gene signatures generated from the human pNF scRNAseq data in the tumor-enriched area indicated by the red box (Fig. 2A and Fig. 6B). The SCL cells and macrophages are enriched and co-localized within the selected area, as visualized by deconvolution scores of SCP-like and MAC-2 signatures (Fig. 6C, D). We also explore the expression of *SPP1*, *CD44*, *NES*, and *ERBB3* (Fig. 6E–H). The turquoise circles show representative spots where SCP-like markers are enriched, and the yellow circles indicate where MAC-2 markers are enriched across Fig. 6. As expected, SPP1 is dominantly expressed in the SCP-like and MMAC-2 spots (Fig. 6E), with co-localized expression of *NES*, *ERBB3*, and *CD44* (Fig. 6F–H). These findings provide further evidence of SPP1-CD44 autocrine and paracrine signaling within the TME.

## Discussion

SPP1 signaling plays multifaceted roles in modulating the inflammatory TME and promoting tumor progression, especially via the SPP1-CD44 axis. The inflammatory signature-enriched molecular subtypes have been revealed in the trascriptome-based subtyping of LGGs and GBMs with *NF1* mutation^51,52^, reflecting biological connections between the *NF1*-related tumors and inflammatory phenotype. This study mechanistically characterized the dynamics of SPP1 during NF1 tumorigenesis and progression using a mouse model, single cell and spatial transcriptomics data.

**Fig. 7:**
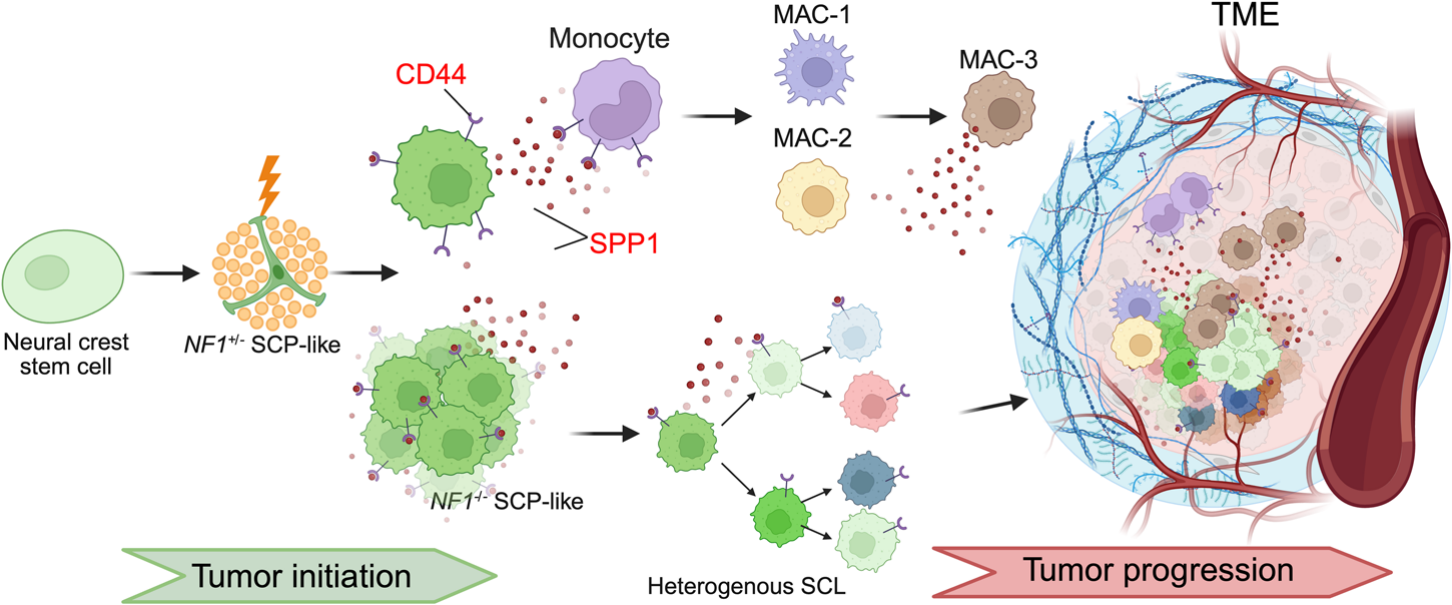
The natural history of SPP1 signaling in NF1 tumorigenesis.

*NF1* loss-of-function in early SCL can disrupt normal myelination, generate chronic damage, and trigger a sustained inflammatory response to the nervous system^53–55^. It is known that sciatic nerve damage, such as a partial transection, promotes NF1 tumorigenesis in mouse models^56^. SPP1’s functions in scar and fibrotic tissue remodeling enhance the accumulation of extracellular matrix components during chronic inflammation within the TME^57,58^. The mild but chronic damage resulting from tumorigenesis can keep recruiting immune and helper cells to the environment. The prolonged inflammation can create a tumor-promoting microenvironment by promoting particular immune populations, especially the alternatively activated macrophages^59,60^. While these macrophages are essential for effective tissue repair, their aberrant activation within the TME can have strong effects on immunoediting and inadvertently promote tumorigenesis through different mechanisms^61^. The macrophages secrete a range of growth factors and cytokines, including TGFβ, that can foster angiogenesis, promote tumor cell migration, and influence SCLs, monocytes, VSMC/Per and T cells, which may lead to the cold immune microenvironment in MPNSTs^62^. A mechanism of SPP1 dynamics in NF1 tumorigenesis is shown in Fig. 7. It is possible that the SPP1 from macrophages can serve as an additional driving force to malignant phenotypes during NF1-associated tumor progression. Meanwhile, additional analysis of SPP1-CD44 signaling should be performed on sporadic or non-NF1-associated MPNSTs before generalization of this axis.

SPP1 from NES + SCP-like tumor cells in the pre-tumor stage indicates that SPP1 signaling is among the early tumorigenesis events, which is not only essential for immune cell recruitment but also maintains the potential of tumor-initiating cells. CD44 expression in the SCP-like tumor cells completes this autocrine loop at the initiating stage, which is supported by the significant survival extension of *cisNf1*^*+/-*^*;Trp53*^*+/-*^*;Spp1*^*-/-*^ (Fig. 1B). The shifting of the SPP1-CD44 axis from autocrine to paracrine during pNF formation and progression emphasizes the essential function of macrophages in TME immunoediting. Recent studies also highlight SPP1 as a prognostic marker for lung cancer and breast cancer^14,45,63^. The maladaptive SPP1-CD44 axis is reinforced during the transformation from pNF to MPNST, suggesting this signaling can also function as an indicator for NF1 tumor progression.

More interestingly, we revealed a dedicated SPP1-secreting macrophage population, MAC-3. Further characterization of MAC-3 may serve as a key to understanding the macrophage programming along tumor progression and provide new targets for treatment. The expression pattern of SPP1 by tumor-initiating cells and macrophages may synergize to create a supportive environment that facilitates the survival of tumor cells and enhances continuous monocyte infiltration and polarization (Fig. 7). This working model paves the way for the SPP1-CD44 axis-targeted treatment strategy for NF1-associated tumors.

## Methods

### Bulk RNA sequencing analysis and solid sarcoma survival analysis

The bulk sequencing data from 54 different NF1 tumors were originally generated by the NF Therapeutic Acceleration Program. The whole dataset is standardized by CBioPortal^35^, and SPP1 expression was retrieved for comparison.

### Mouse *cisNf1*^*+/-*^*;Trp53*^*+/-*^;*Spp1*^*-/-*^ model and survival curve

*Spp1*^*-/-*^ strain was purchased from Jax lab with strain#:004936, and then bred with *cisNf1*^*+/-*^*;Trp53*^*+/-*^ configuration to obtain the *cisNf1*^*+/-*^*;Trp53*^*+/-*^;*Spp1*^*-/-*^. The survival of the mouse was documented and compared with the survival data of *cisNf1*^*+/-*^*;Trp53*^*+/-*^. The mice were maintained according to the approved protocol AUA7457 by the Institutional Animal Care and Use Committee of the Medical College of Wisconsin. Log-rank-based survival analysis was performed with GraphPad Prism v10.

### Single-cell RNA sequencing analysis

Human PN and MPNST scRNA-seq datasets were downloaded from GEO (GSE179043). Mouse PN scRNA-seq datasets were downloaded from GEO (GSE181985). Seurat (version 5.1.0) was used for quality control, normalization, dimensionality reduction, and clustering^64^. Cells with fewer than 250 UMIs and genes expressed in fewer than 10 cells were filtered out. Doublet detection was performed using scDblFinder (version 1.18.0)^65^ with default parameters using the cluster-agnostic method. Doublets were filtered out, and cells with over 20% mitochondrial genes were filtered out. Harmony (version 1.2.1)^66^ was used for batch effect removal to integrate multiple datasets. Seurat’s SCTransform was used for normalization, scaling, and identification of highly variable genes. Highly variable genes were subsequently used to perform linear dimensionality reduction using principal component analysis (PCA). Unsupervised clustering and non-linear dimension reduction using Uniform Manifold Approximation and Projection (UMAP) were performed to visualize the clusters.

Cell type identities were assigned to clusters based on expression of canonical markers.

### Data integration and batch correction

Harmony (version 1.2.1) was used to integrate scRNA-seq datasets and correct for batch effects. SCTransform normalization was performed independently on each sample to integrate. Highly variable features across samples were identified using Seurat’s function SelectIntegrationFeatures. The samples were merged then linear dimensionality reduction was performed using PCA. Harmony was used to correct PCA embeddings for batch effects, generating batch-corrected embeddings. The Harmony embeddings were then used for unsupervised clustering and non-linear UMAP dimensionality reduction.

### Intercellular signaling analysis

CellChat (version 2.1.2)^67^ was used to evaluate intercellular signaling in the scRNA-seq datasets. For analysis of individual datasets, the Seurat object with annotated cell types was converted to a CellChat object, and then intercellular signaling networks were computed. For a comparative analysis of intracellular signaling between tumor types or conditions (human PN vs. human MPNST and control mouse vs. mouse PN), the respective datasets were integrated using Harmony and jointly clustered. Then, they were split back into two Seurat objects using the Seurat function, SplitObject. The two Seurat objects were used to create CellChat objects, which were used to infer intercellular signaling independently. The two CellChat objects were merged and then used for quantitative comparative analysis.

### scRNA-seq pseudotime analysis

Pseudotime analysis was performed using Monocle3 (version 1.3.7)^68^. Seurat (version 5.1.0)^64^ was used for quality control, normalization, dimensionality reduction, clustering, and clusters were visualized using UMAP. The Seurat objects were converted to CellDataSet objects, transferring expression data, UMAP cell embeddings, and cluster identities. The principal graph was constructed from the reduced dimension space to represent developmental trajectories. A principal graph node was selected as the root node, then cells were ordered by pseudotime based on position in the principal graph.

### Spatial transcriptomics

Spatial sequencing data (10X Visium) of plexiform neurofibroma were downloaded from GEO (GSM3484259)^6^. Giotto Suite (version 4.1.5)^69^ was used for quality control, normalization, dimensional reduction, and clustering. Spots with fewer than 500 detected genes and genes detected in fewer than 10 spots were filtered out. The expression data was normalized for total library size and scaled by a factor of 6000. Highly variable genes were computed and used to perform linear dimensionality reduction using PCA. Non-linear dimensionality was performed using UMAP, and unsupervised clustering was performed using Leiden clustering. Cell type deconvolution was performed using Spatial Dampened Weighted Least Squares (DWLS) within Giotto Suite^70^. The human PN scRNA-seq data were used as the reference data. A signature matrix was created using the normalized expression data, cluster identities, and signature genes as computed using the FindAllMarkers function in Seurat. The signature matrix was then used to perform DWLS deconvolution.

### Survival analysis

Survival data were queried through the Cbioportal using TCGA TCGA Soft Tissue Cancer. Source data from GDC and generated in Jul 2024 using ISB-CGC BigQuery tables. Log-rank test is applied. SPP1 normalized expression is used. SPP1 > 0.8 is defined as high expression, and SPP1 < −0.8 is low expression.

### Statistical analysis

Data presentation and statistical analyses are described in the figure legends. The statistical significance was set at *p* < 0.05. Normality was validated for all Student’s *t*-tests; otherwise, nonparametric Mann–Whitney *U*-tests or Kruskal–Wallis tests were applied. When evaluating Spearman’s correlations, the R package was utilized to compute a *p*-value test for noncorrelation.

## Supplementary information

Supplementary Information

## Data Availability

Human PN and MPNST scRNA-seq datasets can be downloaded from GEO (GSE179043). Mouse PN scRNA-seq datasets can be downloaded from GEO (GSE181985). The intermediate data from the analyses are also available upon request. Otherwise, data is provided within the manuscript or supplementary information files.

## Code availability

This study was completed with R version 4.4.1 (2024-06-14 ucrt). We didn’t generate any new R package or specific algorithms. All R code can be accessed upon request.
