# Supplementary material for: Natural history of SPP1 signaling in NF1 tumors
Source: NPJ Precis Oncol. 2025 Oct 6;9:320. doi: 10.1038/s41698-025-01078-2 (PMC12501028; doi:10.1038/s41698-025-01078-2)
Supplement: Supplementary file 1 — Supplementary Information [file 41698_2025_1078_MOESM1_ESM.pdf]

Supplementary Figure

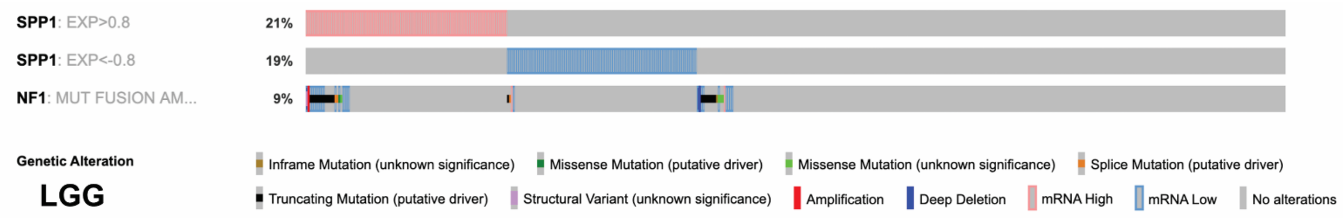

Supplementary Figure 1: Oncoprint Mutation analysis of the *NF1* gene in *SPP1*^{high} and SPP1^{low} LGG patients. Data are curated and visualized by cBioportal.

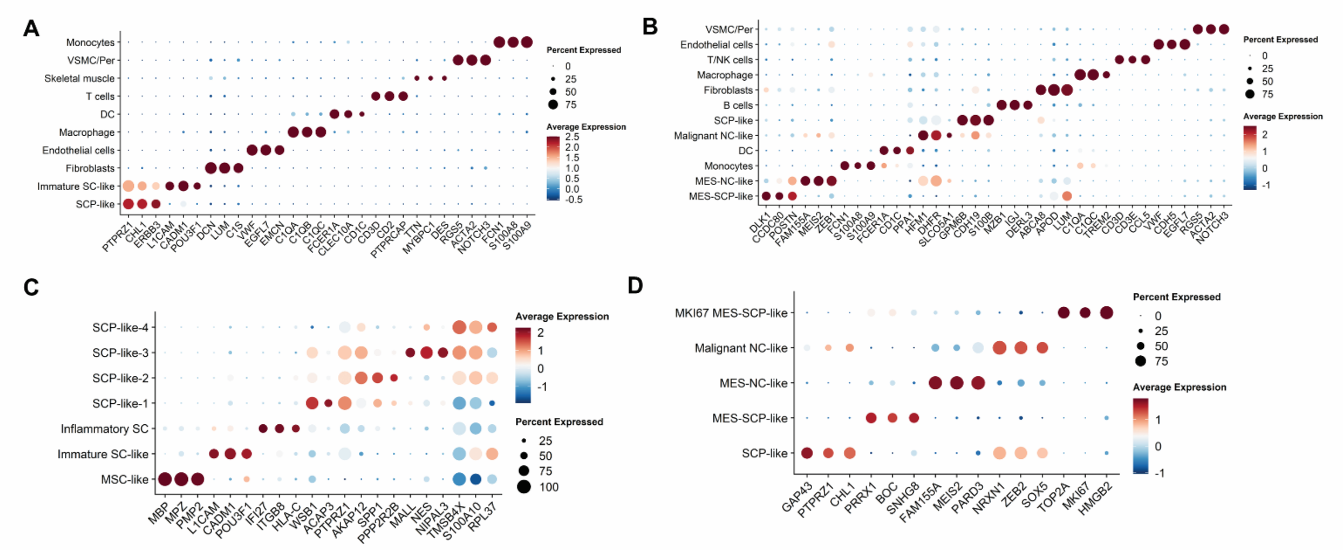

Supplementary Figure 2: Top signatures of identified clusters. (A) human pNF scRNAseq analysis. (B) SCL populations in pNF. (C) human MPNST scRNAseq analysis. (D) SCL populations in MPNST.

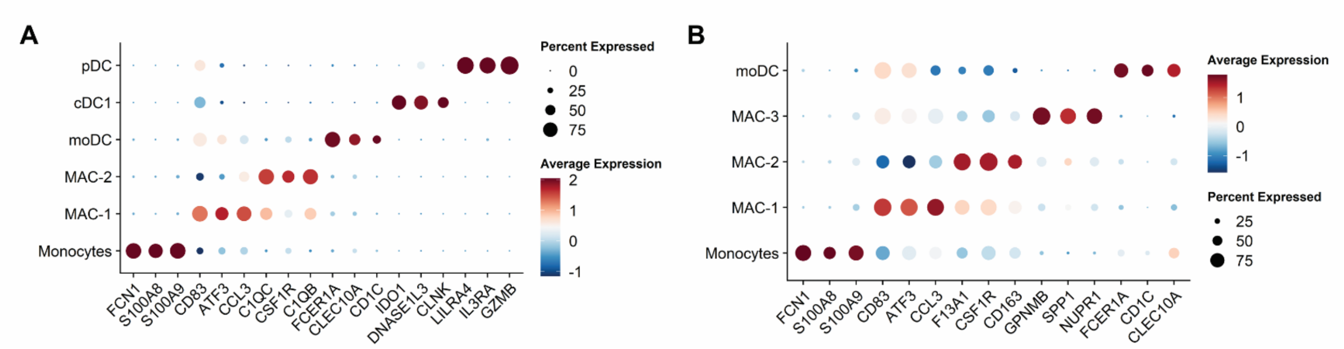

Supplementary Figure 3: Top signatures of identified clusters of myeloid populations in (A) human pNF and (B) human MPNST scRNAseq analysis.
